# A qualitative appraisal of stakeholder reactions to a tool for burden of disease–based health system budgeting in Ghana

**DOI:** 10.3402/gha.v9.30448

**Published:** 2016-05-30

**Authors:** John Koku Awoonor-Williams, Margaret L. Schmitt, Janet Tiah, Joyce Ndago, Rofina Asuru, Ayaga A. Bawah, James F. Phillips

**Affiliations:** 1 Regional Health Directorate, Ghana Health Service PMB, Bolgatanga, Ghana; 2 Swiss Tropical and Public Health Institute, Department of Epidemiology and Public Health, Basel, Switzerland; 3 Faculty of Sciences, University of Basel, Basel, Switzerland; 4 Mailman School of Public Health, Columbia University, New York, NY, USA

**Keywords:** burden of disease, budgeting, evidence-based planning, health systems, qualitative appraisal, Ghana

## Abstract

**Background:**

In 2010, the Ghana Health Service launched a program of cooperation with the Tanzania Ministry of Health and Social Welfare that was designed to adapt Tanzania's PLANREP budgeting and reporting tool to Ghana's primary health care program. The product of this collaboration is a system of budgeting, data visualization, and reporting that is known as the District Health Planning and Reporting Tool (DiHPART).

**Objective:**

This study was conducted to evaluate the design and implementation processes (technical, procedures, feedback, maintenance, and monitoring) of the DiHPART tool in northern Ghana.

**Design:**

This paper reports on a qualitative appraisal of user reactions to the DiHPART system and implications of pilot experience for national scale-up. A total of 20 health officials responsible for financial planning operations were drawn from the national, regional, and district levels of the health system and interviewed in open-ended discussions about their reactions to DiHPART and suggestions for systems development.

**Results:**

The findings show that technical shortcomings merit correction before scale-up can proceed. The review makes note of features of the software system that could be developed, based on experience gained from the pilot. Changes in the national system of financial reporting and budgeting complicate DiHPART utilization. This attests to the importance of pursuing a software application framework that anticipates the need for automated software generation.

**Conclusions:**

Despite challenges encountered in the pilot, the results lend support to the notion that evidence-based budgeting merits development and implementation in Ghana.

## Introduction

Widespread commitment by governments to decentralize planning has arrived at a time when budgetary pressures on health sectors are mounting. This situation has generated international interest in developing tools to support officials in engaging in budgeting and financial planning in ways that shape priorities according to evidence of actual need. Despite increased efforts and commitment for strengthening health systems, many countries lack evidence-based budgeting capacity. This problem is especially prominent in resource-constrained programs in sub-Saharan Africa (SSA) where evidence-based planning is needed most (1, 2). The process of allocating resources across competing programs and interventions occurs at all levels of the health system, involving a range of players and impacting differently on different segments of populations (3). Decision-making processes are complex, oftentimes *ad hoc*, with decisions grounded in political considerations or past budgetary decisions rather than actual need (4). Shifts in priorities often lack transparent criteria for governing the process of change (5).

These inadequacies are exacerbated by disease-specific vertical programs, each with separate systems that overburden health personnel (2, 6). In response, some countries have implemented policy reforms to arrest this situation, including revisiting the primary health care strategy (7). For example, recent health budget system reforms in Ghana have led to the decentralization of discretionary budgeting responsibilities to the district level, despite a lack of attention to equipping managers with tools for this important new planning, budgeting, and monitoring responsibility. Previously, allocation of resources at the district level was based on past expenditure schemes that were driven largely by vertical programs rather than the needs of the entire health system (8). Persistent health equity challenges still exist in many parts of Ghana due to poor planning and a failure to link resource allocation to the burden of disease (9).

Although budgeting has been decentralized, tools for facilitating the planning process have been lacking. There is a need for mechanisms that are guided by well-reasoned criteria that facilitate the planning process and increase transparency. In response, many types of criteria for priority setting in health have been developed, including the cost effectiveness of an intervention, the severity of disease, and the concept of burden of disease analysis (10–12). While there is widespread consensus that systems thinking is needed, debate exists on which criteria should be the most important in setting priorities (5). For example, research conducted in Uganda that assessed health workers’ perceptions of the development of criteria found that many considered the severity of disease as the leading criterion to follow (5). Others contend, however, that burden of disease analysis, which measures ill health in terms of population morbidity and mortality, should be used to assist the process of priority setting and enable planners to promote interventions targeting the most prevalent diseases (10). To support resource allocation practices, a variety of tools are being developed to aid planners in more effectively utilizing these criteria. However, evidence suggests that, due to low perceptions of creditability, such facilitative tools are rarely used in low-income settings (5, 13). Owing to growing interest in improving the access, motivation, and utilization of decision-making tools for the allocation of limited resources, several types of health care prioritization tools have emerged in recent years, many of which are intended to serve the needs of low-income countries, including the Marginal Budgeting for Bottlenecks tool developed by UNICEF and the World Bank (14, 15), the Johns Hopkins University's Lives Saved Tool (16), and the World Health Organization's WHO-CHOICE (Choosing Interventions That Are Cost-Effective) tool (17, 18). More contextualized country-specific models have also been developed, including the Essential Health Research approach in Cameroon and South Africa, the Combined Approach Matrix in Malaysia and Pakistan, and the PlanRep tool in Tanzania (19).

As yet, Ghana lacks a system for district managers to analyze and allocate resources in this manner (20, 21). Rather, most resource allocation is conducted at the district level and based on previous expenditure schemes, which are grounded on prior budgets and projections of programmatic needs that are based on conjecture rather than evidence. The need for budgetary system reforms increased with the introduction of Ghana's sector-wide approach and associated policies, which expanded discretionary budgetary authority to district authorities (22). District health management team (DHMT) facilities were advised in 2004 by the Policy, Planning, Monitoring and Evaluation (PPME) division of the Ghana Health Service (GHS) to develop needs-based budgets. However, an analysis of the 2004 plans by the established budget management centers indicated that the needs-based budgets and proposals far exceeded the available government funding allocated for health, by nearly US$275 million. In addition, it was found that many of the activities proposed by the districts did not target the major causes of Ghana's burden of disease, thus illustrating how such plans would fail to capitalize on the possible gains that would be associated with the utilization of cost-effective and proven interventions. This appraisal indicated that simply increasing funding to health directorates would not adequately address the serious health challenges in the districts. Rather, health planners and national directors needed not only to increase the funding allocated to districts but also to provide tools that would enable the districts to more effectively allocate resources based on need, as indicated by the burden of disease patterns in a given district.

To address this need for systems development, an initiative known as the *Ghana Essential Health Intervention Project* (GEHIP) developed and implemented a qualitative and quantitative district health planning tool, referred to as the *District Health Planning, Analysis, and Reporting Tool* (DiHPART). This tool was developed in collaboration with the Tanzanian Ministry of Health and Social Welfare and the University of Dar-es-Salaam Computing Centre. Based on the logic of the Tanzanian system (PlanRep), DiHPART adapted Tanzania's use of the burden of disease analysis to Ghanaian budgetary requirements, basing its financial profiling on integrated mortality data from the demographic sentinel surveillance Navrongo Health Research Centre. DiHPART aimed to provide district managers guidance on resource allocation, with a focus on the gaps in service delivery, especially those with the greatest potential for reducing maternal and under-five mortality. These objectives are critical for Ghana as the country continues to strive toward achieving the Millennium Development Goals.

DiHPART aims to assist DHMTs to improve their planning capabilities through the utilization of evidence-based indicators and to support the allocation of resources based on reliable quality data. DiHPART also seeks to improve managers’ ability to align their budgets and plans with the districts’ priority needs (8). In addition, the tool intends to enhance district management capacity in the preparation of plans that effectively take into consideration cost-effective interventions that can tackle the health priorities of that district with their budget ceiling, analyze plans against actual outcomes, and compare planned targets against actual performance.

### The financial system and DiHPART

The DiHPART tool was introduced as a pilot in September 2010 in three GEHIP study districts in the Upper East Region (UER): Bongo, Builsa, and Garu-Tempane. The introduction of this tool embraced certain assumptions such as the need for health workers to sustain existing budgeting procedures, while structuring all use of flexible funds according to a model for optimizing investment impact on the burden of disease.

**Fig. 1:**
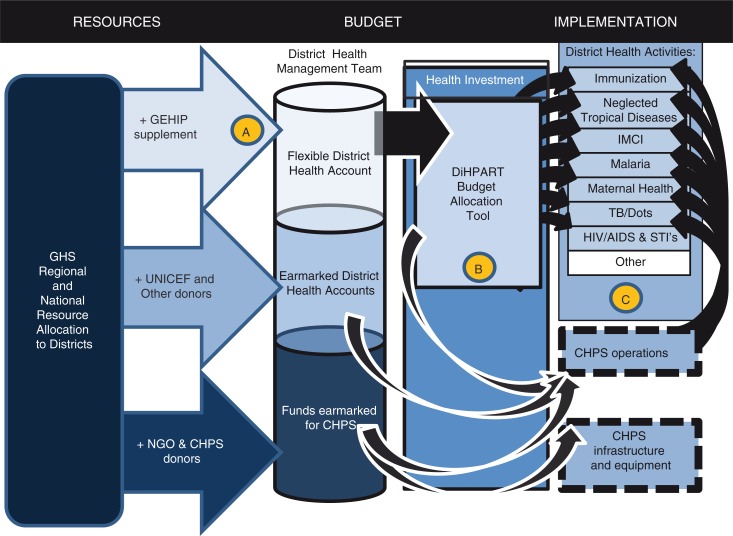
A model for the posited impact of DiHPART on the burden of disease.

Figure 1 portrays the operational assumptions underlying the DiHPART system. As the figure shows, primary health care is supported by Government of Ghana resources that are either earmarked or flexible, with much of the earmarking related to personnel rules that obligate the GHS to prioritize budgetary planning on existing staff salaries and benefits. However, donors contribute to earmarked budget allocations. UNICEF, in particular, is a major supporter of primary health care development. Ghana's Community-Based Health Planning and Services (CHPS) has no budget line for initial start-up costs, apart from a modest annual budget for launching new zones each year. However, NGOs and other donors sometimes invest in construction or equipment costs. DiHPART is predicated on the assumption that there are flexible funds from the GHS that GEHIP could augment with $0.85 per capita for 3 years, with the tool used to optimize this investment (Fig. 1, ‘A’). Because CHPS is a strategy that offsets the burden of childhood disease, DiHPART was assumed to be consistent with the allocation of flexible resources to CHPS start-up costs (Fig. 1, ‘B’). Taken as a set of investments and activities, the combined configuration of investment was posited to improve health and survival, most prominently the health and survival of vulnerable children (Fig. 1, ‘C’).

The objective of this study was to evaluate the design and implementation processes (technical, procedures, feedback, maintenance, and monitoring) of the DiHPART tool in northern Ghana. We consulted with stakeholders in DiHPART implementation, gauged their views of resource allocation process (as illustrated in Fig. 1) and then sought opinions on the use of the tool during the pilot, its ease of use, usefulness, influence on budget priorities, and challenges with design. We sought to utilize the information we received to chart a course for DiHPART implementation and development in the future.

## Methods

### The setting

The UER of Ghana, where the DiHPART tool was implemented, borders both Togo and Burkina Faso and is comprised of 13 rural districts. The UER is the poorest of Ghana's 10 regions (23). However, due to concentrated scale-up efforts, the UER has the highest coverage of the CHPS initiative, Ghana's national primary health care program, than any other region (24, 25). About a third of the population was covered by doorstep CHPS services at the start of pilot activities (25). The UER is known to be the most impoverished region of Ghana and a setting where resource constraints are profoundly challenging to local health authorities.

### Study design

A qualitative systems appraisal was employed as a means to gain understanding of the experiences of trainers, managers, and developers with the design, implementation, and utilization of the DiHPART tool. This included 12 members of DHMTs responsible for utilizing the tool, including district health information officers, district accountants, and district directors. In addition, four members of the national GHS PPME division who were integral in the design and development of the tool were interviewed, including the technical engineering leads and training facilitators. Lastly, four members of the GHS staff at the regional level responsible for training and monitoring the usage of the tool were also interviewed, including the regional health information officers and GEHIP program coordinators. Participants were purposefully drawn from the sample of district health directorates utilizing the tool and stratified according to experience with the DiHPART tool and exposure to training activities to provide a range of understanding among facilities in using the tool.

### Data collection and analysis

A total of 20 in-depth interviews (IDIs) were conducted, with respondents representing the three types of officials who are engaged in routine budgeting in the Ghana Health Service system: 1) national planners responsible for inter-regional budgeting (four officials); 2) regional officials who are responsible for coordinating district financial planning (four officials); and 3) 12 DHMT members, of whom four were district directors of health services for each GEHIP study district and eight were other members of the DHMT who were responsible for planning and financial management in the four GEHIP districts. In addition, we consulted with an internationally known software engineer, as well as participating Ghana Health Service software technicians.

All IDIs were conducted in English, audio-recorded, and transcribed. The questions covered how regional- and district-level health staff utilized the DiHPART tool, if at all; their perceptions on its ease of use and the usefulness and appropriateness of the training and technical support systems; ways to improve the design and roll out of the DiHPART tool to better address the needs of district-level health planners; whether using the tool had influenced the budget priorities assigned to different interventions and in what way; and the greatest challenges with the design, implementation, and usage of the DiHPART tool. The transcripts were reviewed and key themes were identified by three researchers using deductive content analysis methodology (26). Practical experience, institutional documentation, and organizational history were all used to inform and guide this process. A codebook was developed based on the predominant thematic categories that emerged from the data. These include reactions to the design of the tool, perceptions of training procedures, opinions on the benefits and drawbacks of the tool, and the perceived impact of DiHPART. The transcripts from the IDIs were coded using the NVivo software package. Sample codes used included *utilization challenges*, *impact benefits*, *impact negative consequences*, and *teamwork*. Several transcripts were double-coded to ensure inter-coder reliability at the onset of coding activities. All data were reviewed systematically by a team of researchers to ascertain the predominant themes.

### Ethical considerations

This study was approved by the Institutional Review Board for the Protection of Human Subjects at both the Navrongo Health Research Centre and Columbia University. All study participants were notified of the study purpose and provided informed consent prior to the interview.

## Results and lessons learned

A total of 10 challenges were defined by respondents, comprising three general domains of system limitations. The technical design of the tool was associated with 1) systems integration, 2) systems design dysfunction, 3) systems architectural dysfunction, and 4) systems inflexibility. The implementation process domain was associated with three additional sets of limitations: 5) training problems, 6) participant computer literacy limitations, and 7) staff turnover problems. Finally, the organizational context for systems change was a domain associated with 8) leadership challenges, 9) pilot fatigue, and 10) incompatibility of the system output with the decision-making context. The study participants provided strategic guidance on these domains and topics, together with potential solutions for resolving the identified challenges and improving future re-engineering of the tool. Discussions also explored the broader challenges related to the organizational context of the health system, including reasons for staff resistance to change and strategies that could address problems.

### Technical challenges

As is commonly observed when new technologies are introduced, a range of technical challenges were identified that hindered users’ utilization of the tool. Both users and the engineers who developed the tool described in detail these issues and their recommendations for improvement.

1) *Systems integration*. For a computer system to function effectively, its interface with other essential functions represents a critically important element of effective systems design.

A key limitation of the current DiHPART prototype is the lack of appropriate systems linkages to existing government financial reporting systems. Ministry of Finance payroll reforms (IPPD) introduced in 2001 included major modifications to government personnel salary structures. This update resulted in an immediate disconnect between the DiHPART tool and the revised IPPD policies. For example, sector budget templates have changed, and at present the new procedure of the Ministry of Finance (MoF) is incompatible with the DiHPART algorithm. In order to both complete the government-mandated reporting requirements and utilize DiHPART, participating DHMTs were required to complete both financial planning and reporting practices independently. This problem generated critical commentary by users who perceived DiHPART as an imposition of ‘double work’ owing to redundant data entry. Furthermore, annual district planning and reporting requirements involved utilization of a MoF-mandated software product known as the *Activate Template*. Because this reporting tool does not synchronize with DiHPART, DiHPART was perceived to be a stand-alone program without relevance to the national financial management system. A national policy maker explained:

Administratively, the challenge was that because that was something that we were hoping to do in future when we scale it up, rather than how does it integrate into the national planning tool? And then also do another entry into the national planning tool, as required by the Ministry of Finance. So it’s kind of a double work

Integration of DiHPART with the MoF-developed Microsoft Access–based Activate software component of the Activate Template system required continuous update information from MoF developers that was lacking, complicating DiHPART development. This problem was compounded by a MoF decision to migrate its software off the Activate Access-based platform to an Oracle product known as *Hyperion*. In theory, software bridges can be developed, but the changes that were instituted complicated the integration of the DiHPART software into MoF-compatible technology. Without sophisticated bridging systems, Oracle-based software is incompatible with the MS Windows–based software that was driving the DiHPART application.

2) *Systems design dysfunction*. Systems integration challenges were compounded by DiHPART's design as a stand-alone software program that lacked Internet or server connectivity. This absence of connective linkages constrained user access to the tool, imposed technical complications that hampered software updating and file sharing procedures, and enhanced virus vulnerability. DiHPART was initially intended for usage on a shared desktop computer at each District Health Directorate, a measure designed to enhance collective usage by DHMT members and that was deemed to be appropriate for the introduction of non-Internet-based software. It is noteworthy that Internet connectivity during the initial deployment of the tool remained low in the pilot districts; thus, this software limitation impeded the use of DiHPART as a collective tool. Both staff preferences for using the tool on their personal computers and the high frequency of computer viruses that this security risk incurred impaired the effective functioning of the system. The process for updating and transferring updated DiHPART files between personal computers and the communal GHS desktop computer with external storage devices (namely USB drives) was found to be the main mode for virus transfer between devices and as such was responsible for debilitating viruses and subsequent operating system crashes. The DiHPART tool could be readily copied onto additional devices and personal laptops. DHMT staff members typically preferred to run DiHPART software on their personal laptops, owing to the mobility and familiarity of personal devices. However, this fragmented approach to DiHPART access complicated the process of merging files that had been resident on personal devices with the central files of the DHMT office. Moreover, the use of personal computers by some DHMT members was associated with the perception that the DiHPART tool was the personal property of individuals who had the requisite personal equipment and skills. As one regional health manager noted:

Because the program was sitting on their computers, then they will be more involved …. So when it's on the Health Information Officer's [computer], then because he is the one in charge of that he uses it more and then the district directors also copied it on their laptops, so they were using it more.

3) *Systems architecture dysfunction*. Study participants often noted that to mitigate these challenges DiHPART needed to be reengineered into a web-based program, enabling broader use by all members of the team. Engineers who reviewed the system proposed a process for transitioning DiHPART to a web-based design that would facilitate the introduction of technical updates needed to keep operations abreast of shifting national standards. Furthermore, web-based capabilities can mitigate challenges associated with updating, sharing, and merging files between individuals and devices, while enhancing virus protection. Both national and regional participants described the benefits of shifting DiHPART to a web-based platform, explaining

If it is web-based, then most of the difficulties that the DHMTs are facing will be phased out, and centralized on, and they wouldn't have to be importing and exporting and copying files and then consolidating them.

We should be thinking of something that is web-based; it shouldn't be stand-alone that you would have to go there [district directorate], so you can imagine somebody leaving all the way to Garu to go and resolve an issue on DiHPART, while it could be resolved remotely.

A web-based program would also facilitate the provision of routine remote technical support, which could be particularly advantageous in rural and distant localities. However, if DiHPART were shifted to a web-based platform, the GHS would be required to improve the Internet access capabilities of all regional health directorates and all DHMTs in Ghana. Sustained access to the Internet is not yet fully developed in many localities of Ghana.

4) *System inflexibility*. The technical recommendations of pilot project users attest to the need for a software redesign that is an application framework rather than a closed system. Budgeting and disease modeling will change in the future, just as change has occurred in the past. However, if every modification requires a complete systems redesign, the DiHPART concept will be challenged by change of any kind. Software innovations in recent years have responded to the need for automating the generation of code revision (27). If this approach were rigorously imposed on DiHPART, software would be developed that automates the generation of a DiHPART system from parameters that are imposed by technicians, without requiring costly and complicated re-engineering support from developers. Change in the software system that arise from changes in the accounting system or the underlying model for translating strategic action into burden of disease outcomes could be anticipated in ways that would enable the next version of DiHPART to automate updates. Trained developer capacity would be required to support system operation, as is now the case, but engineering flexibility could facilitate adaptation of the system to changing needs. This software concept represents an important required feature of DiHPART that is not adequately addressed by the current system. A re-engineering of DiHPART could anticipate the ‘cloud capability’ needs, computing architecture, automated software generation, and user-oriented features that would facilitate change (28).

### The implementation process

The implementation process can be described as a combination of strategies and practices aimed at introducing and supporting the adoption and utilization of the DiHPART tool by district health staff and regional-level supervisors. This includes training procedures, management and supervisory practices, and other external factors that can impact such processes. The DiHPART tool was introduced through an initial orientation and training in which both the district directors and district accountants were invited.

5) *Training problems*. The training procedures for introducing the tool were found to be inadequate in both frequency and breadth of content. Furthermore, sessions were considered to be too short in duration, generally lasting only a day, and delivered under the premise that sessions were ‘Refreshers’ building from existing basic competencies. In fact, such capabilities were often lacking. Initial trainings involved attendance by three members of each DHMT, including the positions of district director, district accountant, and health information officer. However, the tool was designed for utilization and input by the entire DHMT team, which is generally comprised of seven or eight members, including broader roles such as disease control officer, public health nurse, and health promotion officer. Some participants noted that making the initial trainings exclusive in attendance diminished the collective ownership and responsibility of the tool. As one DHMT member noted:

If all the units could be involved, so that in case one is not there, you can call on any member to come and support, key in certain information. Because it shouldn't be like only three people, what if the three people are not there. So one must think that many members of the DHMT should be involved and even some of the district's heads.

6) *Computer literacy limitations*. Training was pursued on the assumption that basic computer skills were in place. However, issues with low baseline computer literacy were cited as an initial barrier to usage, resulting in heavier reliance on more computer-conversant members of the team and considerable resistance to system utilization among some trainees.

7) *Staff turnover problems*. A high frequency of staff transfers in addition to perceptions of the role of DiHPART as being for data extraction rather than utilization purposes may have also contributed to weak ownership. The high staff transfer rate was a serious impediment to tool adoption. Staff transfers, a routine and frequent practice among Ghana Health Service personnel, resulted in the constant rotation of health workers unfamiliar with the tool and its technical requirements. Follow-up trainings, which occurred once a year, were cited as inadequate for handling the constant influx of untrained personnel. As one national facilitator indicated:

The transfer – that was very harmful to the system and there were a lot of transfers that occurred. There were some people who were trained but transferred out, which virtually collapsed the whole system. In one or two of the districts it happened like that; they took out those who were trained and those who came knew nothing DiHPART.

### Organizational contextual challenges

8) *Leadership challenges*. The high frequency of staff transfers in addition to perceptions of the DiHPART's role being for data extraction rather than utilization purposes may have also contributed to weak ownership. Not all members of the DHMTs perceived DiHPART as a tool for supporting their own work, but rather see it introduced due to the time constraints of data entry and existing and routine data extraction expectations. One regional supervisor indicated this disconnect, alluding to the need to

…. help them to do proper budgeting at their level and they should own whatever output they derived from the software and it shouldn't be like somebody somewhere wants them to do the entries and will come for the report later on.

9) *Pilot fatigue*. Managerial skepticism about DiHPART was grounded to some extent in perceptions of pilot fatigue, with indications that some DHMT members failed to embrace DiHPART due to wariness about its sustainability, for as one manager noted, it is not being used

…. because it is another pilot, and we have not fully adopted it or that kind of thing; we don't use it mostly very often.

10) *Incompatibility of the system output with the decision-making context*. DiHPART visualizes the burden of disease implications of budget scenarios, under the assumption that DHMTs have the authority and capability to reallocate resources according to categories of decision-making that data visualization portrays. In fact, the visualization criteria were so heavily borrowed from Tanzania that their relevance to strategic planning in Ghana was compromised. Discussion of budgeting as portrayed by DiHPART was inconsistent with operational decision-making options that DHMTs could actively embrace. DiHPART displays bar charts that compare the pattern of disease burden that is consistent with estimated patterns relative to the pattern implied by proposed systems investments. Classes of outcomes displayed, however, represent a mixture of activities such as integrated management of childhood illnesses (IMCI) and disease syndromes, such as malaria or HIV/AIDS. Activity classes are too broad to define optimal resource decisions. IMCI, for example, involves both facility-based investment and community-based care. Community care, in turn, is comprised of volunteer activities and the activities of paid nurses. Lumping all such investments into a single indicator constrains the decision-making contribution of DiHPART. A new model for classifying categories of actions and outputs is needed.

A challenge related to the compatibility of DiHPART with the organizational context concerns the emergence of multisectoral financial planning. Figure 1 posited a framework for the flow of resources and the role of automated planning in the rationalization of priority setting. The GEHIP program, however, has developed a multisectoral approach to leadership development, in response to national programming for the decentralization of revenue sharing. In this policy framework, budgeting and finance for the health sector can involve partnership with the development sector. In response, the project developed a program of community engagement that has had the direct benefit of implementing program functioning of CHPS with backing from local politicians and development partners. This approach responds to the broad-based policy shift in Ghana toward revenue pooling at the periphery into common funds that are multisectoral. The DiHPART approach, to be effective, must adapt to this decision-making reality. The allocation of district flexible resources involves a variety of development options, each with potential impact on well-being. For the health sector to contribute to the process of decision-making about these funds, approaches limited to the burden of disease may be appropriate, but only if options for resource allocation include investments that district political leaders and development partners and officials can embrace. The start-up costs of CHPS is an example of a component of DiHPART that is inadequately addressed. Moreover, visualization tools in the DiHPART system are not yet focused on decision-making options that officials can consider.

**Fig. 2:**
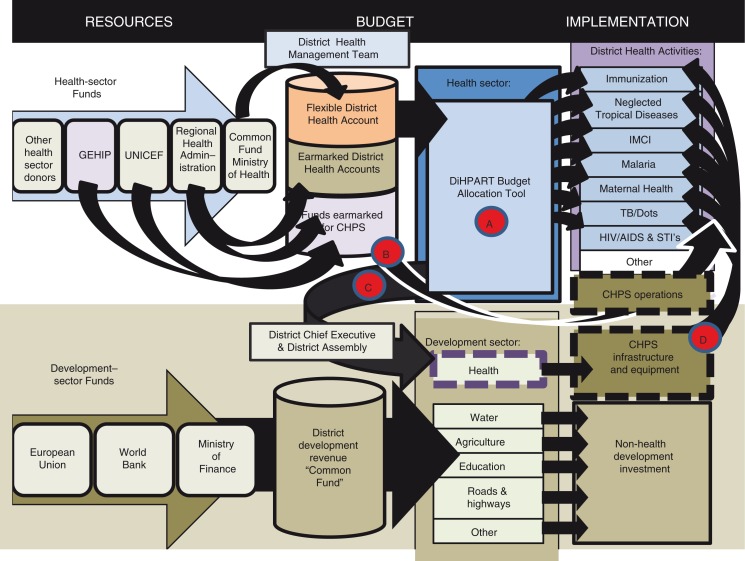
The complex resource decision-making environment implied by the context of a multisectoral common fund arrangement.

Figure 2 illustrates the conceptual challenge of resource allocation in a multisectoral environment. As the figure shows, health sector flexible financing is a minor factor in the more general resource allocation environment. First, as the diagram shows, direct financing of the health sector is complex, with little flexibility vested in the common fund (Pathway A, Fig. 2). Some latitude for district financing is associated with Regional Health Administration flexibility, but the amounts available are marginal to the overall level of financing. Earmarking is important and can convey more flexibility than accounting systems connote. For example, UNICEF is a major donor of motorbikes, clinical equipment, and essential supplies. These critical resources support the system in general, enabling integrated services to be provided and CHPS implementation to progress even though resources are targeted on specific items or needs. However, the underlying assumption of DiHPART that flexibility can be a resource for rational data-driven planning is unrealistic. Once the bare essentials are addressed, no remaining funds exist for DiHPART-informed priority setting.

The lower panel of Fig. 2 portrays a level of complexity that is realistic but missing from the framework of Fig. 1. If interchange between the health sector and the development sector is well planned, well informed with evidence, and grounded in community-engaged support for health development, then DiHPART, at least in theory, could be a resource for demonstrating to grassroots politicians and officials the survival potential of appropriate investment in health (Pathway C, Fig. 2). This investment could foster CHPS implementation through the allocation of resources that have no budget line in the health sector but are crucial to getting CHPS started. Once even the most makeshift and temporary community facility exists, GHS staff are available to fill essential posts. Budget lines are available for resource planning for running CHPS, but start-up costs are not available. GEHIP could provide a visualization tool for advocating catalytic investment in starting CHPS operations.

## Discussion

### DiHPART results visualization

As a consequence of the 10 themes noted in the course of the IDIs, it was apparent that DiHPART could not be sustained as a health sector budgeting tool and was not at all compatible with multisectoral planning: At no point in the discussion sessions was there any mention of using DiHPART results to communicate health sector priorities to district chief executives or other local officials. Moreover, respondent skepticism of the utility of the tool for routine budgeting was expressed in some form by all study participants. Nor was there discussion of shifts in the operation of the program that were attributed to DiHPART data visualization. As yet, the system lacks the format and content of visualized outcomes of health investment that would be appropriate for motivating intersectoral exchanges about the benefits of health investment to district populations or even structural resource allocation within the health sector. For example, if CHPS is to be supported by incremental development investment, then DiHPART health visualization tools should include bar graphs for relevant development decision-making options and DHMT operational planning or other displays that show the burden of disease or life-saving potential of investment in CHPS implementation or other service strategies. Models for such a display have yet to be configured, but their design is feasible given the existence of relevant data from the Navrongo Health Research Centre. Clearly, a new DiHPART tool is needed, not only because objective data-driven tools are welcomed by participants in the present study and because data resources exist for the requisite modeling task, but also because the context for DiHPART application is shifting in ways that require new strategies and a new system.

Our conclusion that a ‘DiHPART-2’ is needed, despite the problems that were noted, is grounded in the general respondent consensus that DiHPART had a beneficial impact on critically important aspects of workflow operations. Such benefits included enhancing collaboration and communication between DHMT positions, improving planning procedures, and promoting greater planning transparency within DHMTs. DiHPART was an instigator for greater interaction between DHMT roles for planning processes and budgetary discussions, a process that in the past was typically conducted by a select few individuals (generally the district directors and district accountants). As one regional supervisor noted:

It had worked very well in that it would bring everyone on board, because there was no way one single person could sit and use the tool. Everybody at the DHMT in a way had an input into the system, so in a way it would have improved a lot of teamwork at the district level.

Improving planning processes was also an identified area in which DiHPART was found to impact DHMT operations. DiHPART served as a mechanism for aiding planning discussions, with a clear focus on the utilization of evidence. Chief planning and decision-making responsibilities in DHMTs are generally designated to district directors, whose decisions are subject to personal intuition and other external determinants; thus such practical guidance was considered as imperative. DiHPART was able to provide practical guidance on how money was being spent and programs delivered in relation to the burden of the disease realities of that particular district. Visualizations were claimed as useful in guiding DHMT-based discussions, especially in promoting a greater sense of transparency in decision-making processes. This clarity was noted to occur on both the area of spending and disease burdens, and was also identified as a means for enhancing directors who were provided with the tool.

The DiHPART case represents a promising approach to health development with positive outcomes. However, experience also attests to the unrealized potential of the system. If DiHPART were a continuing activity with a standing software team, links between users and developers, and a process for systems adaptation over time, the ideas that underlie DiHPART could have been more effectively developed, positioning the system for scale-up.

## Conclusions

It is evident that there is a clear need for improved budgetary decision-making tools to enhance the efficiency and effectiveness of health systems, especially in low-income settings. However, such tools must be both developed with and accepted by their intended users. With the proliferation of mobile health applications (mHealth application) around the world, there is mounting evidence of the importance of piloting procedures that highlight the complexities of the health system for which they are intended to operate and adapt technology to the realities that piloting can identify. Valuable systems learning emerged from the DiHPART pilot, demonstrating the value of systems thinking as integral to the process of improving budgeting decision-making. However, piloting requires a total systems approach that includes coordination of new applications with all relevant sectors and units within the health and financial systems to ensure that the introduction of new tools streamlines the workloads and facilitates the decision-making of intended users. Furthermore, the introduction of a new technology is an on-going process and cannot be considered as a singular event. Bringing about change needs to be an iterative process, requiring continual trainings and updates, like other existing training programs already in place within the health sector. If the recommendations for improvement can be integrated into an improved version of the DiHPART tool, there is great potential for it to improve district-level health operations and ultimately the health of the population they serve.

## Paper Context

With decentralization policies that are introduced throughout Africa, district managers often have expanded responsibilities for planning and budgeting. However, software supporting this function is often poorly designed, and suitable for centralized budgeting only. In response to this problem, the Ghana Health Service collaborated with the Tanzania Ministry of Health and Social Welfare to transfer and adapt a burden of disease–based planning tool to Ghana's needs. This paper describes the transfer process and the tool that emerged.

## References

[1] DeSavigny D, Binka F (2004). Monitoring future impact on malaria burden in sub-Saharan Africa. Am J Trop Med Hyg.

[2] Chretien JP, Burkom HS, Sedyaningsih ER, Larasati RP, Lescano AG, Mundaca CC (2008). Syndromic surveillance: adapting innovations to developing settings. PLoS Med.

[3] Kapiriri L, Norheim O (2004). Criteria for priority setting in health interventions in Uganda. Exploration of stakeholders’ values. Bull World Health Organ.

[4] Ham C (1997). Priority setting in health care: learning from international experience. Health Policy.

[5] Kapiriri L, Norheim OF, Martin DK (2009). Fairness and accountability for reasonableness. Do the views of priority setting decision makers differ across health systems and levels of decision making?. Soc Sci Med.

[6] Chen LC, Evans T, Anand S, Boufford JI, Brown H, Chowdhury M (2004). Human resources for health: overcoming the crisis. Lancet.

[7] Frenk J (2009). Reinventing primary health care: the need for systems integration. Lancet.

[8] Nyonator F, Ofosu A, Osei D, Schmitt ML, Awoonor-Williams JK, Phillips JF (2015). District Health Planning and Reporting Tool (DiHPART): transferring a tool for district level evidence-based planning and budgeting from Tanzania to Ghana.

[9] Couttolenc BF (2012). Decentralization and Governance in the Ghana Health Sector. http://dx.doi.org/10.1596/978-0-8213-9589-9.

[10] Baltussen R, Niessen L (2006). Priority setting of health interventions: the need for multi-criteria decision analysis. Cost Eff Resourc Alloc.

[11] Cookson R, Dolan P (1999). Public views on health care rationing: a group discussion study. Health Policy.

[12] Kapiriri L, Arnesen T, Norheim OF (2004). Is cost-effectiveness analysis preferred to severity of disease as the main guiding principle in priority setting in resource poor settings? The case of Uganda. Cost Eff Resourc Alloc.

[13] Youngkong S, Kapiriri L, Baltussen R (2009). Setting priorities for health interventions in developing countries: a review of empirical studies. Trop Med Int Health.

[14] Fryatt R, Mills A, Nordstrom A (2010). Financing of health systems to achieve the health Millennium Development Goals in low-income countries. Lancet.

[15] Knippenberg R, Soucat A, Vanlerberghe W (2003). Marginal budgeting for bottlenecks: a tool for performance based planning of health and nutrition services for achieving Millennium Development Goals.

[16] Victora CG (2010). LiST: using epidemiology to guide child survival policymaking and programming. Int J Epidemiol.

[17] Adam T, Lim SS, Mehta S, Bhutta ZA, Fogstad H, Mathai M (2005). Cost effectiveness analysis of strategies for maternal and neonatal health in developing countries. BMJ.

[18] Edejer TT (2003). Making choices in health: WHO guide to cost-effectiveness analysis.

[19] DeSavigny D, Munna G, Mbuya C, Mgalula L, Machibya H, Mkikima S District Health Expenditure Mapping: a budget analysis tool for Council Health Management Teams (No. 1). Dar es Salaam: 2001.

[20] Hill K, Lopez AD, Shibuya K, Jha P, AbouZahr C, Anderson RN (2007). Interim measures for meeting needs for health sector data: births, deaths, and causes of death. Lancet.

[21] Sullivan DF (1971). A single index of mortality and morbidity. HSMHA Health Rep.

[22] Cassels A (1997). A guide to sector-wide approaches for health development: concepts, issues and working arrangements.

[23] Ghana Statistical Service (2010). Population & housing census report.

[24] Nyonator FK, Awoonor-Williams JK, Phillips JF Scaling down to scale up: accelerating the expansion of coverage of community–based health services in Ghana.

[25] Awoonor-Williams JK, Sory EK, Nyonator FK, Phillips JF, Wang C, Schmitt ML (2013). Lessons learned from scaling up a community-based health program in the Upper East Region of northern Ghana. Glob Health Sci Pract.

[26] Elo S, Kyngäs H (2008). The qualitative content analysis process. J Adv Nurs.

[27] Peischl B, Ferk M, Holzinge A (2014). The fine art of user-centered software development. Software Qual J.

[28] Johnson CM, Johnson TR, Zhang JA (2005). A user-centered framework for redesigning health care interfaces. J Biomed Informat.

